# Generation of Higher-Order Poincaré Beams with Polarization States Varying Along the Propagation Direction Based on Dielectric Metasurfaces

**DOI:** 10.3390/nano15070478

**Published:** 2025-03-22

**Authors:** Kaixin Zhao, Teng Ma, Manna Gu, Qingrui Dong, Haoyan Zhou, Yuantao Wang, Wenxin Wang, Chuanfu Cheng, Chunxiang Liu

**Affiliations:** School of Physics and Electronics, Shandong Normal University, Jinan 250014, China; zhaokaixin_1999@163.com (K.Z.); mateng_mt1320@163.com (T.M.); gumanna1996@outlook.com (M.G.); zhouhaoyanrock@163.com (H.Z.); wangyuantao0210@163.com (Y.W.); 19854190974@163.com (W.W.); chengchuanfu@sdnu.edu.cn (C.C.)

**Keywords:** metasurface, vector beams, longitudinal manipulation, spatial partitioning

## Abstract

Vector beams (VBs) with longitudinally varying polarization states provide a new dimension for light field manipulation, and promote the advancements of related areas such as optical metrology, longitudinal depth detection, and classical and quantum communications. In this study, we propose a half-wave plate dielectric metasurface based on a spatial partitioning method, realizing the longitudinal manipulation of the polarization states of higher-order Poincaré (HOP) beams by changing the elliptical polarization state of the incident light and selecting the appropriate propagation distances. The metasurface is composed of two sub-metasurfaces, and the two sets of a-Si:H meta-atoms are uniformly arranged on concentric rings of different radii with an equal interval. The propagation and Pancharatnam–Berry phases are utilized to construct the axicon and helical phase profiles. As a result, two sub-metasurfaces, respectively, generate the first- and second-order VBs with longitudinally varying polarization states. The polarization states of generated VBs correspond to points on different meridians of nth-order HOP spheres from the south pole to the north pole. The consistency between the theoretical and simulated results demonstrates the feasibility and practicability of the proposed method. This study provides an innovative strategy to extend the modulation of light fields from two-dimensional to three-dimensional space.

## 1. Introduction

A vortex beam is an electromagnetic wave carrying orbital angular momentum, and it has a helical phase front and usually shows a doughnut-like intensity distribution [1,2,3]. Owing to the mode orthogonality between vortex beams with different topological charges, vortex beams can greatly increase information capacity and have potential applications in optical communication [2,4]. Moreover, vortex beams can also be used for applications such as quantum information [5,6], and trapping and rotating particles [7]. Polarization is an inherent property of light. In recent years, in addition to spatially homogeneous polarization states of light such as linear, circular, and elliptical polarization, vector beams (VBs) with spatially inhomogeneous polarization distributions have attracted widespread attention. VBs have a wide range of applications in classical fields, such as metrology [8], imaging [9], particle acceleration [10], etc. With inseparable coupling characteristics of spatial and polarization degrees of freedom, VBs, as a new type of light resource, also have important applications in the quantum field, such as in teleportation [11], and quantum key distribution [12]. In the last decades, numerous methods have been proposed for generating VBs, which can be realized using different bulky interferometers [13,14,15]. Additional optical devices such as lenses were generally required for realizing the focusing of VBs. However, these traditional optical devices limit the flexibility of manipulation of VBs. Recently, the emergence of artificial materials has attracted increasing attention, providing a prominent method for realizing high-performance optics.

In recent decades, with sub-wavelength scale thickness and the ability to control the polarization, amplitude, and phase of light fields, artificial material metasurfaces composed of dielectric meta-atoms have gradually developed into an emerging field of scientific research, and have broad application prospects in various fields, such as metalenses [16,17,18], vectorial full-color holography [19], structured beam generators [20,21], and manipulation and measurement of quantum states [22], etc. Among the numerous applications, the use of metasurfaces to generate diverse VBs is a research hotspot due to the spin-orbit coupling effect of light with the meta-atoms. Earlier designs of metasurfaces focused on generating radially and azimuthally polarized VBs by directly manipulating the polarization of nanostructures, while this method is inconvenient manipulation and the type of generated VBs is relatively simple. Subsequently, Yue et al. proposed a novel strategy for designing metasurfaces to generate VBs by superposing two orthogonal circularly polarized (CP) vortices based on the principles of higher-order Poincaré (HOP) spheres [23,24], and then this method has been widely used to generate diverse VBs, including perfect HOP beams [25], and multichannel VBs [26,27,28].

Nevertheless, previous investigations have mainly focused on manipulating the polarization distribution only on a single transverse plane. Recently, with in-depth research, the modulation of optical fields has progressively expanded from two-dimensional (2D) to three-dimensional (3D) space, and many achievements have been made to realize the continuous control of the polarization states along the propagation direction. Yang et al. utilized two axicon phases and combined them with incident-polarization to realize arbitrary manipulation of the polarization states of the generated HOP beams [29]. Huang et al. demonstrated the use of a birefringent all-dielectric metasurface to generate a Bessel beam with longitudinally varying polarization state [30,31]. Furthermore, Yao et al. realized longitudinal manipulation from homogeneous to inhomogeneous polarization states of light fields [32,33], and generated multichannel VBs with continuously changing polarization states along the propagation direction [34,35]. Moreover, Luo et al. proposed a method of spin-decoupled spatial partitioning to realize the continuous and periodical switching of polarization state and generate high-order vector optical field with continuous variation from second- to 10th-order [36,37]. However, these studies mainly concentrated on longitudinally manipulating VBs with different linear polarization states. There is rarely research that longitudinally manipulates VBs on points of different meridians of an nth-order HOP sphere using only a single metasurface.

In this study, we proposed a half-wave plate (HWP) dielectric metasurface using spatial partitioning method, realizing the longitudinal manipulation of the polarization states of HOP beams by changing the elliptical polarization states of the incident light and selecting the appropriate propagation distances. The polarization states of generated VBs correspond to points on different meridians from the south pole to the north pole on nth-order HOP spheres. The metasurface is composed of two sub-metasurfaces, and the two sets of a-Si:H meta-atoms are uniformly arranged on concentric rings of different radii with an equal interval. The propagation and Pancharatnam–Berry (PB) phases are utilized to construct the phase profiles for imparting on the light fields of each sub-metasurface. Specifically, the propagation phase is utilized to construct one axicon phase, and the transmitted light field is deflected to form a long longitudinal depth for longitudinal manipulation. The PB phase is used to construct helical phases, thereby generating a pair of orthogonal CP vortices carrying topological charges of the same absolute value but opposite signs. For the two sub-metasurfaces, the topological charges of helical phases are set to 1 and 2, respectively. Moreover, the PB phase is used to construct the other axicon phase for making two orthogonal CP vortices carry axicon phases with opposite signs owing to the chirality dependence of the PB phase. As a result, the orthogonal CP vortices are deflected differently, that is, the longitudinal depths of orthogonal CP vortices undergo small reverse shifts in the propagation direction, leading to a phase difference between the orthogonal CP vortices. Therefore, the phase difference between the left and right circular polarization (LCP and RCP) vortices varies with propagation distance *z* within the longitudinal depth, realizing the longitudinal manipulation of phase difference of generated VBs. As a result, two sub-metasurfaces, respectively, generate first- and second-order VBs with longitudinally varying polarization states in the different propagation distance. In addition, by changing the elliptical polarization states of the incident light, the weights of the LCP and RCP vortices can also be manipulated, and the polarization state of the generated VB evolves from one pole to the other along the meridian on the HOP sphere. Therefore, based on the proposed design, longitudinal manipulation of polarization states of VBs on different meridians of an nth-order HOP sphere can be realized by controlling the elliptical polarization state of the incident light and selecting an appropriate propagation distance under the combination of propagation and PB phase. We first performed theoretical analysis for the generation of the vector field with polarization states of longitudinal variations. Then, a simulation was conducted using finite-difference time-domain (FDTD) software. Obviously different from the previous studies, HOP beams with variable order and arbitrary elliptical polarization along the propagation direction can be generated based on a single metasurface in our study, rather than being limited to generating VBs with linearly polarized states on the equator of an HOP sphere [36,37] or single-order HOP beams using a single metasurface [29] along the propagation direction. The feasibility and practicability of the proposed method have been verified in both theory and simulation.

## 2. Principle of Metasurface Design

### 2.1. Overview of Principle

**Figure 1:**
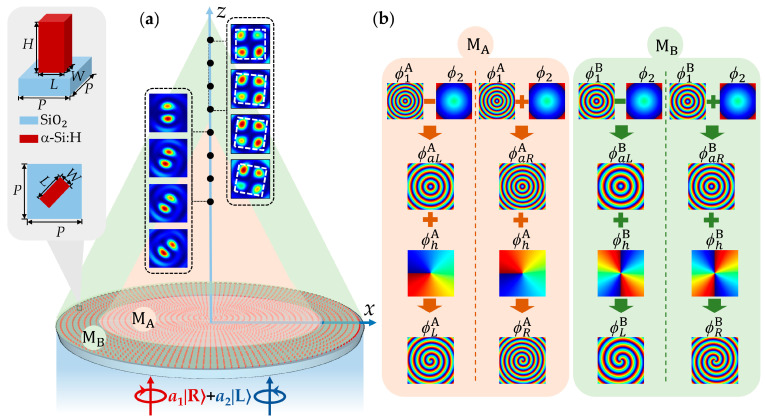
Schematic of principles and methods of metasurface design. (**a**) Schematic illustration of longitudinal modulation of generated VBs with the metasurface. The inset depicts the side and top views of the meta-atoms of the metasurface. (**b**) The phase profiles of sub-metasurfaces M_A_ and M_B_ in the proposed metasurface design, respectively.

Figure 1a shows a schematic of the longitudinal manipulation of VBs generated by an HWP metasurface under the elliptically polarized light illumination. The proposed metasurface comprises two sub-metasurfaces M_A_ and M_B_ distinguished by subscripts A and B. The sub-metasurfaces M_A_ and M_B_ are composed of two sets of meta-atoms arranged on concentric rings of different radii. The meta-atoms are rectangular nanopillars of a-Si:H on a SiO_2_ substrate with length *L*, width *W*, height *H*, and lattice period *P*, where the height *H* of the meta-atoms is constant, and the lattice period *P* is the increment of the radii of adjacent rings. Their side and top views are shown in the left panel of Figure 1a. Under the illumination of linearly polarized (LP) light, the transmitted light field consists of a pair of orthogonal CP vortices (**|***L*⟩ and **|***R*⟩), which superpose to form VBs within a long longitudinal depth. Notably, the topological charges of helical phases for the two sub-metasurfaces are set to 1 and 2, respectively. Therefore, the first- and second-order VBs are generated, and the *x*-component intensity patterns of the generated VBs under LP illumination are shown in Figure 1a. The polarization states of VBs evolve along the propagation direction, which correspond to points on the equator of the HOP sphere. By adjusting the elliptical polarization of the incident light and choosing a different propagation distance *z* behind the metasurface, VBs with continuously varying polarization states can be generated along the propagation direction. The polarization states correspond to the points from one pole to the other on different meridians of the HOP sphere.

In order to longitudinally manipulate the superimposed optical field, Figure 1b gives the detailed phase design of the transmitted light field at *z* = 0 for each sub-metasurface by combining the propagation and PB phases. In the phase design of sub-metasurface M_A_, firstly, the propagation phase is utilized to constructed axicon phase profile $\phi_{1}^{A}$ with the smaller radial period $d_{1}^{A}$, with the result that the transmitted light field is deflected to form a long longitudinal depth. Then, the PB phase is used to construct the helical phase profile $\phi_{h}^{A}$ to generate two orthogonal CP vortices with topological charges of the opposite signs and same absolute value. In addition, the PB phase is used to yield another axicon phase profile $\phi_{2}$ with the radial period $d_{2}$, and owing to the chirality dependence of the PB phase, the longitudinal depths of two orthogonal CP vortices undergo smaller reverse longitudinal shifts, relative to the position of the original longitudinal depth. By combining the propagation phase and PB phase, the axicon phases of the LCP and RCP vortices can be expressed as $\phi_{aL}^{j}=\phi_{1}^{j}-\phi_{2}$ and $\phi_{aR}^{j}=\phi_{1}^{j}+\phi_{2}$, respectively, where subscript *j* = A or B, respectively, represent two sub-metasurfaces M_A_ and M_B_. Therefore, within the longitudinal depth of the two orthogonal CP vortices, the phase difference between the LCP and RCP vortices varies with the propagation distance *z*. The polarization states of the generated VBs continuously change along the propagation direction, corresponding to the points on the equator of the HOP sphere under LP light illumination. In addition, by changing the polarization state of the incident light, the weights of the LCP and RCP vortices can also be manipulated, and the polarization states of VBs correspond to the points from one pole to the other on the HOP sphere. For the sub-metasurface M_B_, the topological charge of the helical phase is different from that of sub-metasurface M_A_. Therefore, based on the different phase design of the two sub-metasurfaces, sub-metasurfaces M_A_ and M_B_ can sequentially generate first- and second-order VBs with longitudinally varying polarization states with the increase in the propagation distance. Therefore, under combined modulation of the axicon and helical phases, as well as the incident light with different elliptical polarization states, it is possible to realize longitudinal manipulation of the polarization states of VBs, corresponding to the points from one pole to the other on different meridians of the nth-order HOP sphere.

### 2.2. Theoretical Analysis of Metasurface-Generated Higher-Order Poincaré Beams

Due to form birefringence, the meta-atom is considered as a wave-plate, and the Jones matrix *J*(*x*,*y*) can be written as:

(1) $$J(x,y)=R(-\theta )\left[\begin{array}{cc} \left|T_{x}\right|e^{i\varphi_{x}(x,y)} & 0\\ 0 & \left|T_{y}\right|e^{i\varphi_{y}(x,y)} \end{array}\right]R(\theta ),$$

where *φ_x_*(*x*,*y*), *φ_y_*(*x*,*y*), |*T_x_*| and |*T_y_*| are the propagation phases and the transmission amplitudes, shifting along the two symmetry axes of the meta-atom, respectively. For the selected meta-atoms, |*T_x_*| and |*T_y_*| can be considered nearly constant values and are approximately considered as 1. *R*(*θ*) is the rotation matrix, and *θ* is the orientation angle of the long side relative to the *x*-axis. When the incident lights of LCP and RCP illuminate an HWP meta-atom with |*φ_y_*− *φ_x_*| = *π*, the transmitted light fields can be represented as:

(2) $$J(x,y)|L\rangle =\exp[i\varphi_{R}(x,y)]|R\rangle ,$$

(3) $$J(x,y)|R\rangle =\exp[i\varphi_{L}(x,y)]|L\rangle ,$$

where $|L\rangle ={\left[\begin{array}{cc} 1 & i \end{array}\right]}^{T}/\sqrt{2}$ and $|R\rangle ={\left[\begin{array}{cc} 1 & -i \end{array}\right]}^{T}/\sqrt{2}$ represent unit vectors of LCP and RCP, respectively. The terms on the right-hand sides of the above two equations are cross-polarized components. Based on Equations (2) and (3), the required Jones matrix *J*(*x*,*y*) can be expressed as follows:

(4) $$J(x,y)=\left[\begin{array}{cc} e^{i\varphi_{R}(x,y)} & e^{i\varphi_{L}(x,y)}\\ -ie^{i\varphi_{R}(x,y)} & ie^{i\varphi_{L}\left(x,y\right)} \end{array}\right]{\left[\begin{array}{cc} 1 & 1\\ i & -i \end{array}\right]}^{-1}.$$

Using Equations (1) and (4), the following equations provide a quantitative relationship of the propagation phase and rotation angle of the meta-atom, so the *φ_x_*(*x*,*y*), *φ_y_*(*x*,*y*), and *θ*(*x*,*y*) can be written as:

(5) $$\varphi_{x}(x,y)=[\varphi_{R}(x,y)+\varphi_{L}(x,y)]/2,$$

(6) $$\varphi_{y}(x,y)=[\varphi_{R}(x,y)+\varphi_{L}(x,y)]/2+\pi ,$$

(7) $$\theta (x,y)=[\varphi_{R}(x,y)-\varphi_{L}(x,y)]/4.$$

Based on the basic theory of the transmitted light fields of a single meta-atom mentioned above, we analyze the transmitted light field of a meta-atom under the illumination of elliptically polarized light **|*u***_in_⟩ = *a*_1_**|***R*⟩+*a*_2_**|***L*⟩, which contains components of RCP (**|***R*⟩) and LCP (**|***L*⟩) with the weights *a*_1_ and *a*_2_, respectively. The transmitted light field ***E***(*r*, *α*) can be written as:

(8) $$E(r,\alpha )=a_{1}[e^{i(\phi_{aL}^{j}+\phi_{h}^{j})}]{\left[\begin{array}{cc} 1 & i \end{array}\right]}^{T}+a_{2}[e^{i(\phi_{aR}^{j}+\phi_{h}^{j})}]{\left[\begin{array}{cc} 1 & -i \end{array}\right]}^{T},$$

where (*r*, *α*) represent the polar coordinates, $\phi_{aL}^{j}$ and $\phi_{aR}^{j}$ are the axicon phases of LCP and RCP vortices (*j* = A or B), respectively, and can be written as $\phi_{aL}^{j}=\phi_{1}^{j}-\phi_{2}$ and $\phi_{aR}^{j}=\phi_{1}^{j}+\phi_{2}$, where $\phi_{1}^{j}=-2\pi r/d_{1}^{j}$, $\phi_{2}=-2\pi r/d_{2}$, so the axicon phases $\phi_{aL}^{j}$ and $\phi_{aR}^{j}$ can be further expressed:

(9) $$\phi_{aL}^{j}=-\frac{2\pi r}{d_{L}^{j}};\phi_{aR}^{j}=-\frac{2\pi r}{d_{R}^{j}},$$

where *r* is the radius of the concentric ring, $d_{L}^{j}$ and $d_{R}^{j}$ are the radial periods of the axicon phases of LCP and RCP vortices, and can be expressed as $d_{L}^{j}=d_{1}^{j}d_{2}/(d_{2}-d_{1}^{j})$ and $d_{R}^{j}=d_{1}^{j}d_{2}/(d_{2}+d_{1}^{j})$. The helical phase profile with topological charge 2*σm* is as follows:

(10) $$\phi_{h}^{j}=2\sigma \left(\theta_{0}+m^{j}\alpha \right),$$

where *σ* is the chirality factor of CP light, *θ*_0_ is the initial orientation angle of the meta-atom, and $m^{j}$ is the rotation order. Based on Equations (8)–(10), the output field of the meta-atom under the illumination of incident light with elliptical polarization can be expressed as follows:

(11) $$E(r,\alpha )=a_{1}[e^{i[-2\pi r/d_{L}^{j}-2(\theta_{0}+m^{j}\alpha )]}]{\left[\begin{array}{cc} 1 & i \end{array}\right]}^{T}+a_{2}[e^{i[-2\pi r/d_{R}^{j}+2(\theta_{0}+m^{j}\alpha )]}]{\left[\begin{array}{cc} 1 & -i \end{array}\right]}^{T}.$$

According to Equation (11), *E*(*r*,*α*) represents the superposition of two orthogonal CP vortices **|**L, −2*m^j^*⟩ and **|**R, 2*m^j^*⟩ with the weights of *a*_1_ and *a*_2_, respectively, and their superposition forms VBs within the long longitudinal depth. The LCP and RCP vortices was imparted different axicon phases of $-2\pi r/d_{L}^{j}$ and $-2\pi r/d_{R}^{j}$, indicating that the different deflections are provided for the two orthogonal CP vortices. Therefore, a *z*-component difference Δ*k_z_* of wavevectors is produced between the LCP and RCP vortices, which is related to radial radius *r*. The phase difference $\phi^{j}(r)$ of the LCP and RCP vortices at a specific radius *r* is expressed as follows:

(12) $$\phi^{j}(r)=-\frac{2\pi r}{d_{L}^{j}}-(-\frac{2\pi r}{d_{R}^{j}})=-\frac{2\pi r(d_{R}^{j}-d_{L}^{j})}{d_{L}^{j}d_{R}^{j}}.$$

The relationship between radial radius (*r*) and propagation distance (*z*) can be expressed as *r* = sin*β* · *z*, and the refraction angle *β* satisfies the equation [29] $d_{L}^{j}\sin\beta =\lambda$. Therefore, the relationship between *r* and *z* can be expressed as $r=z\lambda /d_{L}^{j}$. Thus, the phase difference $\phi^{j}(r)$ can be converted into the phase function $\phi^{j}(z)$ related to propagation distance *z* as follows:

(13) $$\phi^{j}(z)=-\frac{2\pi z\lambda (d_{R}^{j}-d_{L}^{j})}{{\left(d_{L}^{j}\right)}^{2}d_{R}^{j}},$$

according to Equation (13), the phase difference of the LCP and RCP vortices originating from Δ*k_z_* is a function related to the propagation distance *z*, indicating that the longitudinal manipulation of polarization states of VBs can be realized by selecting the propagation distance *z* from the metasurface. In addition, the weights of the LCP and RCP vortices can be manipulated by changing the polarization state of the incident light. Therefore, by changing the elliptical polarization state of the incident light and selecting different propagation distances, longitudinal manipulation of polarization states of VBs can be realized, and their polarization states correspond to points from the south pole to the north pole on different meridians of the HOP sphere.

## 3. Simulations and Numerical Analysis

**Figure 2:**
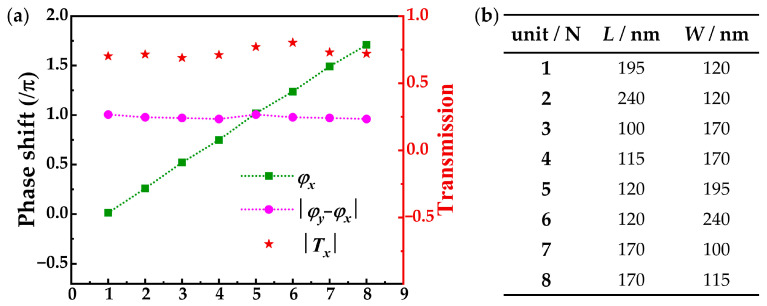
(**a**) The propagation phase *φ_x_*, phase retardation |*φ_y_* − *φ_x_*|, and the *x*-direction transmission amplitude *T_x_* versus the sequential number of HWP meta-atoms (N). (**b**) Dimensions of the eight selected meta-atoms.

To validate the proposed design, we used FDTD software (Lumerical 2020 R2) to perform 2D parameter sweeps over the transmitted light field of a meta-atom and simulate the light fields of the metasurface. We used rectangular nanopillars of a-Si:H with a fixed height of 480 nm. The lattice constant was set to 350 nm. At a wavelength of 800 nm, the refractive index of the meta-atoms was set to 3.744, and the extinction coefficient was set to 0.000. In the parameter sweeps, the side length ranged from 80 nm to 330 nm in steps of 1 nm. Under the illumination of *y*-LP light, the boundary conditions of *x*, *y*, and *z* were set to “Symmetric”, “Anti-symmetric”, and “Perfectly Matched Layer (PML)”, respectively. Similarly, under the illumination of *x*-LP light, the boundary conditions of *x*, *y*, and *z* were set to “Anti-symmetric”, “Symmetric”, and “Perfectly Matched Layer (PML)”, respectively. Therefore, when the meta-atom was illuminated with *x*- and *y*-LP light, the propagation phases *φ_x_* and *φ_y_* of the meta-atoms with different dimensions were obtained. In Figure 2a, we selected eight HWP meta-atoms with the conditions of the phase retardation |*φ_y_*− *φ_x_*| = *π* for metasurface design based on these simulation results. The propagation phase *φ_x_* was uniformly increasing within the range of 0 to 2*π*, and the amplitude *T_x_* for these meta-atoms was close to 1. The dimensions of the eight meta-atoms are shown in Figure 2b.

We designed the metasurface sample using eight selected meta-atoms. The meta-atom located at the metasurface coordinates (*r*, *α*) was selected from these eight HWP meta-atoms, and its dimension and orientation angle were determined by the axicon and helical phase profiles of Equations (9) and (10). The designed metasurface sample was composed of concentric rings with equal intervals of 350 nm, and meta-atoms were uniformly arranged on each ring at interval of 350 nm. The diameter of the metasurface sample was 70 μm. Based on spatial partitioning, the metasurface samples were partitioned into two sub-metasurfaces, labeled M_A_ and M_B_, as shown in Figure 1a. The sub-metasurface M_A_ was composed of meta-atoms on the first to 75th rings with the radial periods of $d_{L}^{A}=1.877$ µm and $d_{R}^{A}=1.553$ µm for the two axicon phases, and the rotation order *m*^A^ of the meta-atoms was set to 0.5, with the result that first-order VBs with longitudinally varying polarization states were generated within the longitudinal depth. Similarly, the other sub-metasurface M_B_ was composed of meta-atoms on rings from 75th to 100th, with the radial periods of $d_{L}^{B}=2.25$ µm and $d_{R}^{B}=1.80$ µm, and the rotation order *m*^B^ was 1, generating second-order VBs with longitudinally varying polarization states within the longitudinal depth. Based on the design of the metasurface sample mentioned above, we simulated the transmitted light field of the metasurface sample using FDTD software. In the simulation, the upper surface position of the substrate of the metasurface was set to *z* = 0, the position of the incident light was set to *z* = −0.8 µm, the wavelength of the incident light was set to 800 nm, and the monitor was set to *z* = 0.8 µm. The span of *x* and *y* of FDTD was set to 72 µm × 72 µm and the *z* span was set from −1 µm to 1.5 µm, which covers the range of the metasurface sample, incident light, and monitor. The mesh type was auto non-uniform and the mesh accuracy was set to 1. Under LP illumination, the phase difference between the LCP and RCP vortices of the first and second-order VBs generated sequentially in the longitudinal direction can vary with the propagation distance *z*, and their polarization states of the generated VBs corresponded to the points on the equator of the HOP sphere. In addition, combining with the incident light with different elliptical polarization states, continuous variation of the weights and phase difference of the LCP and RCP vortices can be realized, and the polarization states of VBs correspond to the points from the south pole to the north pole on different meridians of the nth-order HOP sphere. Specifically, in FDTD simulations, we first simulated the light distribution of the near-field plane for the metasurface sample under different elliptically polarized light illumination, and then projected it onto the far-field plane to obtain the VBs at different propagation distances *z*.

**Figure 3:**
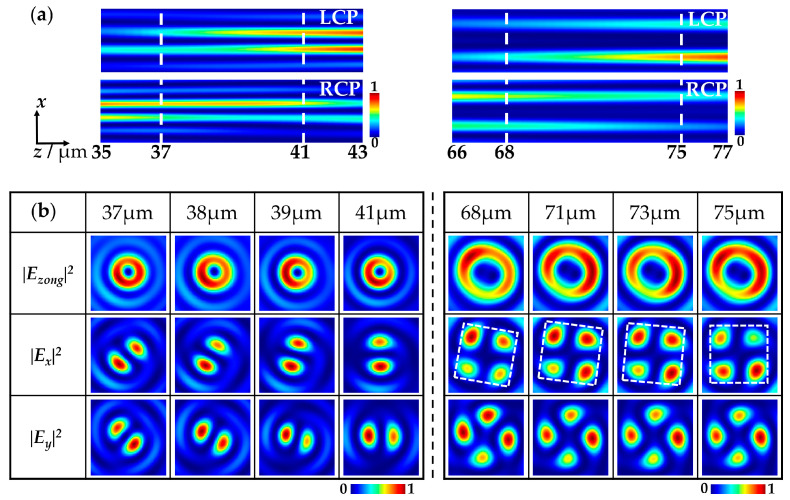
(**a**) Intensity images of the LCP and RCP components of VBs generated by the metasurface sample in the *x*–*z* plane under *x*-LP illumination. (**b**) The intensity images of VBs generated under *x*-LP illumination. The total intensity, *x*-, and *y*-component intensities of the transmitted light field are displayed in rows from the top to bottom. The left to right columns show the intensity patterns of VBs at different propagation distances *z*. The dashed line is used to distinguish the transmitted light fields of sub-metasurfaces M_A_ and M_B_.

Figure 3a shows the simulation results of LCP and RCP vortices on the *x*–*z* plane within the long longitudinal depth under *x*-LP illumination. According to the figure, the intensity of the LCP and RCP vortices were roughly equal in the range of 37 µm to 41 µm and 68 μm to 75 μm, that is, the weights of LCP and RCP vortices is approximately equal to 1:1, so linearly polarized VBs can be obtained within the longitudinal depth. Figure 3b shows the intensity patterns of total and component fields on the *x*–*y* plane at different propagation distances *z*. The title row represents the position coordinates along the propagation direction, and the rows from top to bottom represent the total intensity patterns and the intensity patterns of the *x*- and *y*-components, respectively. Both intensity images of the *x*- and *y*-components have the lobes divided by the maximal dark lines, and the dark lines more quantitively express the linear polarization features of the wavefields. The relationship between the number N of lobes and the topological charge *l* is N = 2*l*, as can be demonstrated by the two-lobe patterns in the left columns of Figure 3b for *l* = +1 and the four-lobe patterns in the right columns of Figure 3b for *l* = +2. We observed that in the range of *z* from 37 μm to 41 μm, the *x*- and *y*-components of the generated VBs had two lobes, and the orientations of the two lobes rotated continuously with distance *z*, indicating that first-order VBs were generated within the longitudinal depth and the phase difference between the LCP and RCP vortices of VBs varied longitudinally. The polarization states of the generated first-order VBs correspond to the points on the equator of the first-order HOP sphere. Similarly, in the range of *z* from 68 μm to 75 μm, VBs with four lobes in the *x*- and *y*-component intensity patterns were generated, and the orientations of the four lobes also rotated continuously with *z*, indicating that the second-order VBs were generated in the longitudinal range and the phase difference between the LCP and RCP vortices also varied continuously along the propagation direction. The polarization states of the generated second-order VBs correspond to the points on the equator of the second-order HOP sphere.

**Figure 4:**
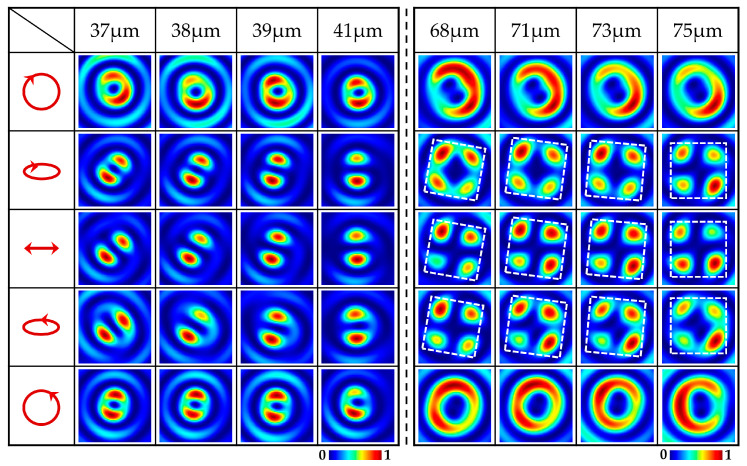
The *x*-component intensity images **|***E_x_***|**^2^ of VBs generated by the metasurface sample under the illumination of different elliptically polarized light. The title column represents the incident light with different elliptical polarization states. The left to right columns display the intensity patterns of VBs at different distances *z*.

We simulated the transmitted light field of the metasurface sample by changing the elliptical polarization of the incident light as shown in Figure 4. The title row represents the position coordinates along the propagation direction, and the rows from top to bottom represent the intensity patterns of the *x*-component under the illumination of different elliptically polarized light. We can observe that the orientations of the two lobes of the *x*- and *y*-components rotated continuously with distance *z*, which was consistent with the conclusion obtained from Figure 3. The patterns of each column show that, when the polarization states of the incident light change from the linear to elliptical and then to circular, the orientations of the lobes in the *x*-component patterns remain unchanged, but their boundaries become blurred. The simulated results demonstrated that the weights of the LCP and RCP vortices of the generated VBs can also be manipulated under the illumination of different elliptically polarized light. Therefore, by changing the elliptical polarization state of the incident light and selecting different longitudinal distances *z*, we can realize the continuous modulation of the polarization state of VBs along the propagation direction, corresponding to points from the south pole to north pole on different meridians of HOP sphere. This is highly consistent with the principle of the metasurface we designed.

## 4. Discussion and Conclusions

Although in our work we only provide detailed theoretical and simulated results, their consistency verifies the feasibility of the proposed design; here, we give some further analysis on the practical feasibility of fabrication and experimental implementation. Regarding sample fabrication, a 480 nm thick a-Si:H dielectric film can be deposited on a fused silica substrate by plasma-enhanced chemical vapor deposition (PECVD) using SiH_4_ and He gases based on the nanostructures obtained from the results of FDTD simulations. The fabricated metasurface sample can be measured in an optical setup similar to reference [38]. LP light with a wavelength of 800 nm is emitted from a laser, and then passes through the HWP and quarter-wave plate (QWP) to became elliptically polarized light. After being attenuated by the attenuator, it illuminates the metasurface sample mounted on a 3D piezo nanometer stage. Subsequently, the VBs generated on the different *z*-planes behind the sample are collected and imaged using a microscope objective, and the intensity patterns of VBs are recorded by the scientific complementary metal-oxide semiconductor detector. Finally, using a polarizer and QWP, the component intensity patterns of the *x*-, 45°-, *y*-, 135°-, LCP, and RCP are obtained, respectively. Thus, sample fabrication and experimental measurement may be practical and feasible.

This method can more effectively achieve longitudinal manipulation of HOP beams under incident light with a wavelength of 800 nm. In the simulation, we selected eight HWP meta-atoms used to construct metasurface at a wavelength of 800 nm, and the size of the meta-atoms is suitable for the specific wavelengths. When the wavelength of the incident light deviates from 800 nm, the meta-atom cannot be considered as an ideal HWP, and its propagation phase cannot satisfy the condition of uniform increase within the range of 0 to 2*π* well, resulting in nonuniform intensity distribution of the VBs generated by the metasurface. In our previous work [39], we have discussed the broadband performance of metasurfaces, in which the VBs generated by metasurfaces samples were simulated with the FDTD method under the illumination of horizontally linear polarization in the wavelength range of 700–900 nm, and the simulation results are best at the wavelength of 800 nm. Furthermore, we have a discussion on the impact of material losses or potential optical aberrations introduced by the metasurface. As shown in Figure 2a, the values of *T_x_* and *T_y_* are in the range of 0.7 to 0.8 instead of being equal to 1 owing to material losses. Thus, the meta-atom cannot be considered as an ideal HWP, which will decrease the quality of the generated HOP beams due to the residual co-polarized components. In our previous work [39], we conducted a study on improving the beams’ quality by eliminating the central bright spot of residual co-polarized components. Cross-talk between adjacent observation planes originating from the transmitted lights of the meta-atoms at different radial coordinates being deflected onto the different observation planes along the propagation direction is inevitable. Although this cross-talk may interfere with the generated HOP beams to some extent, it will not change the fundamental characteristics of the intensity distribution of the HOP beams overall.

In summary, we propose an HWP dielectric metasurface based on a spatial partitioning method, which is composed of two sub-metasurfaces. By changing the elliptical polarization state of the incident light and selecting different longitudinal distances *z*, continuous manipulation of the polarization states of first-order and second-order VBs can be realized along the propagation direction, and their polarization states correspond to points on different meridians from the south pole to north pole on the nth-order HOP sphere. The axicon and helical phase profiles were constructed by utilizing propagation phase and PB phase. Based on the proposed metasurface design, it can be extended to realize longitudinal manipulation of the polarization state of VBs from first-order to an arbitrary higher order. This article provides detailed theoretical and simulated results, and their consistency verifies the feasibility of the proposed design. This study on the longitudinal manipulation adds new degrees of freedom, and opens up new possibilities for optical field manipulation. VBs with longitudinally varying polarization states promote the advancements of related areas such as microfabrication, optical communications, longitudinal depth detection, biomedical applications, material processing, and volume laser machining. The research results are of great significance for the miniaturization of optical devices and the integration of optical systems.

## Data Availability

The data that support the findings of this study are available from the corresponding author upon reasonable request.
